# 4,4′-Dichloro-2,2′-[(3a*R*,7a*R*/3a*S*,7a*S*)-2,3,3a,4,5,6,7,7a-octa­hydro-1*H*-1,3-benzimidazole-1,3-di­yl)bis­(methyl­ene)]diphenol

**DOI:** 10.1107/S160053681003792X

**Published:** 2010-09-30

**Authors:** Augusto Rivera, Diego Quiroga, Jaime Ríos-Motta, Michal Dušek, Karla Fejfarová

**Affiliations:** a Departamento de Química, Universidad Nacional de Colombia, Bogotá, AA 14490, Colombia; b Institute of Physics, Na Slovance 2, 182 21 Praha 8, Czech Republic

## Abstract

Mol­ecules of the the title compound, C_21_H_24_Cl_2_N_2_O_2_, are located on a twofold rotation axis, which passes through the C atom linking the two N atoms. Two intra­molecular O—H⋯N hydrogen bonds were observed. In the crystal, non-classical inter­molecular C—H⋯O hydrogen bonds link the mol­ecules into chains along the *a* axis. The crystal studied was a racemic twin.

## Related literature

For related structures, see: Rivera *et al.* (2009 ▶, 2010 ▶). For uses of di-Mannich bases, see: Mitra *et al.* (2006 ▶); Elias *et al.* (1997 ▶). For the anti­malarial activity of di-Mannich bases, see: Shanks & Edstein (2005 ▶).
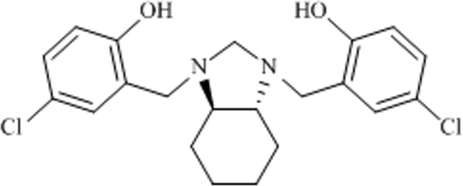


## Experimental

### 

#### Crystal data

C_21_H_24_Cl_2_N_2_O_2_
*M* *_r_* = 407.3
Orthorhombic, 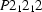

*a* = 5.9529 (2) Å
*b* = 18.3846 (5) Å
*c* = 8.9704 (3) Å
*V* = 981.74 (5) Å^3^
*Z* = 2
Cu *K*α radiation
μ = 3.11 mm^−1^
*T* = 120 K
0.36 × 0.21 × 0.12 mm

#### Data collection

Oxford Diffraction Xcalibur diffractometer with an Atlas (Gemini ultra Cu) detector
Absorption correction: analytical [*CrysAlis PRO* (Oxford Diffraction, 2009 ▶), based on expressions derived by Clark & Reid (1995 ▶)] *T*_min_ = 0.518, *T*_max_ = 0.773
12720 measured reflections
1566 independent reflections
1517 reflections with *I* > 3σ(*I*)
*R*_int_ = 0.027

#### Refinement

*R*[*F* ^2^ > 2σ(*F* ^2^)] = 0.022
*wR*(*F* ^2^) = 0.068
*S* = 1.50
1566 reflections
127 parameters
H atoms treated by a mixture of independent and constrained refinement
Δρ_max_ = 0.11 e Å^−3^
Δρ_min_ = −0.11 e Å^−3^
Absolute structure: Flack (1983 ▶), 615 Friedel pairs
Flack parameter: 0.32 (1)

### 

Data collection: *CrysAlis CCD* (Oxford Diffraction, 2009 ▶); cell refinement: *CrysAlis RED* (Oxford Diffraction, 2009 ▶); data reduction: *CrysAlis RED*; program(s) used to solve structure: *SIR2002* (Burla *et al.*, 2003 ▶); program(s) used to refine structure: *JANA2006* (Petříček *et al.*, 2006 ▶); molecular graphics: *DIAMOND* (Brandenburg & Putz, 2005 ▶); software used to prepare material for publication: *JANA2006*.

## Supplementary Material

Crystal structure: contains datablocks global, I. DOI: 10.1107/S160053681003792X/bt5333sup1.cif

Structure factors: contains datablocks I. DOI: 10.1107/S160053681003792X/bt5333Isup2.hkl

Additional supplementary materials:  crystallographic information; 3D view; checkCIF report

## Figures and Tables

**Table 1:** Hydrogen-bond geometry (Å, °)

*D*—H⋯*A*	*D*—H	H⋯*A*	*D*⋯*A*	*D*—H⋯*A*
C1—H1*a*⋯O1^i^	0.95	2.56	3.3398 (11)	137.58
O1—H1*o*⋯N1	0.91 (2)	1.83 (2)	2.6515 (13)	149.3 (18)

Symmetry code: (i) 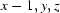.

**Fig. 1.**
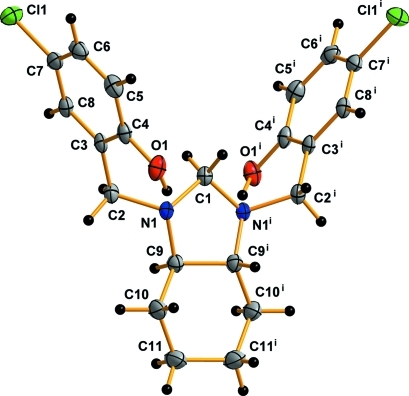
Molecule of the title compound with atom-labeling scheme. Displacement elipsoids are drawn at 50% probability level.

**Fig. 2.**
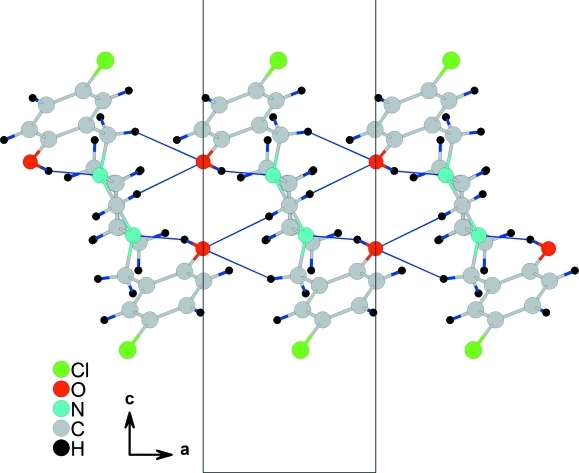
Hydrogen bonding of the molecules of the title compound in a direction.

## supplementary crystallographic information

### Comment

It is interesting to notice that two types of non-classical intermolecular hydrogen bonds of C—H···O and C—H···Cl were found according to crystallographic data. The molecular structure and atom-numbering scheme for (I) are shown in Fig. 1. Its X-ray structure confirms the presence of intramolecular hydrogen bonds between the phenolic hydroxyl groups and nitrogen atoms [N—H, 1.83 (2) Å), whereas the N···O distances [2.652 (2) Å,] is significantly shorter than the corresponding N···O bond in related structures [2.70 (1) Å]. Furthermore the observed C—O bond length [1.354 (2) Å] is considerably shortened in relation to related structures [1.364 (2) Å] (Rivera *et al.*, 2010) and [1.365 (2) Å] (Rivera *et al.*, 2009). This additional H-bonding does not influence the H—O distance, which shows (as a result of unrestrained refinement) a typical separation of 0.91 (2) Å. Thus, these results indicate an increase in hydrogen-bonding strength due to the presence of chlorine atom. In fact, the presence of the chlorine atom favours the formation of weak intermolecular C—H···O interactions between neighboring molecules, which link them into 1-D extended chains along the *a* axis and help to stabilize the chain.

The chains are linked along the c direction by C—H···Cl interactions [2.902 (2) Å]. This interaction involves contacts between an apparently electron deficient aromatic C6—H6 and the chlorine atom from a second molecule. The phenyl group in both molecules lies in an orientation which favours hydrogen bond formation.

In the title compound, C_21_H_24_Cl_2_N_2_O_2_,the asymmetric unit contains one-half of the molecule, which is related to the other half by a twofold rotation axis [symmetry code: - *x*, *y*, -*z*] passing through C1 (Figure 1). Unlike the related structures Rivera *et al.* (2010, 2009), the title compound crystallizes in a different crystal system and it has a chiral space group. The compound is a racemic twin and the absolute structure was determined on the basis of that of the starting amine whose stereochemistry is: *trans*-(*rac*)-1,2-cyclohexanediamine and the chiral centers were not affected when reacted.

### Experimental

Preparation of title compound (I)

A solution of (2*R*,7*R*,11*S*,16S)-1,8,10,17-tetraazapentacyclo[8.8.1.18,17.0^2,7^.0^11,16^] icosane (276 mg, 1.00 mmol) in dioxane (3 ml) and water (4 ml), prepared beforehand following previously described procedures, was added dropwise in a dioxane solution (3 ml) containing two equivalents of *p*-chlorophenol (257 mg, 2.00 mmol) in a two-necked round-bottomed flask. The mixture was refluxed for about 6 h until precipitation of a colourless solid. The resulting solid was collected by filtration, washed with cool methanol and dried under vacuum (yield 30%, m.p. = 490–492 K). Next, the racemic product (100 mg, 0.246 mmol) was dissolved in 5 ml of a 4:1 mixture of chloroform: methanol. Single crystals of title compound (I) suitable for X-ray analysis were grown by slow evaporation of the solvent at room temperature over a period of about 2 weeks in a preferential crystallization (yield 46%). 1H NMR (CDCl_3_, 400 MHz): δ 1.29 (4*H*, m), 1.85 (2*H*, m), 2.04 (2*H*, m), 2.34 (2*H*, m), 3.42 (2*H*, d, J = 14.0 Hz, ArCH_2_N), 3.51 (2*H*, s, NCH_2_N), 4.14 (2*H*, d, J = 14.0 Hz, ArCH_2_N), 6.74 (2*H*, d, J = 8.8 Hz), 6.92 (2*H*, s), 7.10 (2*H*, d, J = 8.8 Hz).

### Refinement

All hydrogen atoms were discernible in difference Fourier maps and could be refined to reasonable geometry. According to common practice H atoms attached to C atoms were nevertheless kept in ideal positions during the refinement. The coordinates of the hydroxyl H atom were refined. The isotropic atomic displacement parameters of all hydrogen atoms were set to 1.2**U*_eq_ of the parent atom.

### Crystal data

**Table:**

C_21_H_24_Cl_2_N_2_O_2_	*F*(000) = 428
*M**_r_* = 407.3	*D*_x_ = 1.378 Mg m^−^^3^
Orthorhombic, *P*2_1_2_1_2	Cu *K*α radiation, λ = 1.54184 Å
Hall symbol: P 2 2ab	Cell parameters from 10371 reflections
*a* = 5.9529 (2) Å	θ = 4.8–62.4°
*b* = 18.3846 (5) Å	µ = 3.11 mm^−^^1^
*c* = 8.9704 (3) Å	*T* = 120 K
*V* = 981.74 (5) Å^3^	Irregular shape, colorless
*Z* = 2	0.36 × 0.21 × 0.12 mm

### Data collection

**Table:**

Oxford Diffraction Xcalibur diffractometer with an Atlas (Gemini ultra Cu) detector	1566 independent reflections
Radiation source: X-ray tube	1517 reflections with *I* > 3σ(*I*)
mirror	*R*_int_ = 0.027
Detector resolution: 10.3784 pixels mm^-1^	θ_max_ = 62.5°, θ_min_ = 4.8°
Rotation method data acquisition using ω scans	*h* = −6→6
Absorption correction: analytical [*CrysAlis PRO* (Oxford Diffraction, 2009), based on expressions derived by Clark & Reid (1995)]	*k* = −21→20
*T*_min_ = 0.518, *T*_max_ = 0.773	*l* = −10→10
12720 measured reflections	

### Refinement

**Table:**

Refinement on *F*^2^	H atoms treated by a mixture of independent and constrained refinement
*R*[*F*^2^ > 2σ(*F*^2^)] = 0.022	Weighting scheme based on measured s.u.'s *w* = 1/[σ^2^(*I*) + 0.0016*I*^2^]
*wR*(*F*^2^) = 0.068	(Δ/σ)_max_ = 0.010
*S* = 1.50	Δρ_max_ = 0.11 e Å^−^^3^
1566 reflections	Δρ_min_ = −0.11 e Å^−^^3^
127 parameters	Absolute structure: Flack (1983), 615 Friedel pairs
0 restraints	Flack parameter: 0.32 (1)
45 constraints	

### Special details

**Table:**

Experimental. CrysAlisPro, Oxford Diffraction Ltd., Version 1.171.33.51 (release 27-10-2009 CrysAlis171 .NET) Analytical numeric absorption correction using a multifaceted crystal model based on expressions derived by Clark & Reid (1995)Physical MeasurementsThe melting point was determined with an Electrothermal apparatus, and it has not been corrected. IR spectrum was recorded as KBr pellets at 292 K on a Perkin-Elmer Paragon FT-IR instrument. NMR spectra were performed in CDCl_3_ at room temperature on a Bruker AMX 400 Advance spectrometer.
Refinement. The refinement was carried out against all reflections. The conventional *R*-factor is always based on *F*. The goodness of fit as well as the weighted *R*-factor are based on *F* and *F*^2^ for refinement carried out on *F* and *F*^2^, respectively. The threshold expression is used only for calculating *R*-factors *etc*. and it is not relevant to the choice of reflections for refinement.The program used for refinement, Jana2006, uses the weighting scheme based on the experimental expectations, see _refine_ls_weighting_details, that does not force *S* to be one. Therefore the values of *S* are usually larger than the ones from the *SHELX* program.

### Fractional atomic coordinates and isotropic or equivalent isotropic displacement parameters (Å^2^)

**Table:**

	*x*	*y*	*z*	*U*_iso_*/*U*_eq_	
Cl1	0.56453 (8)	0.22892 (2)	0.85004 (4)	0.03657 (14)	
O1	0.99889 (19)	0.41865 (6)	0.41066 (13)	0.0278 (3)	
N1	0.59603 (9)	0.44360 (5)	0.29542 (10)	0.0191 (4)	
C1	0.5	0.5	0.39535 (12)	0.0190 (6)	
C2	0.5511 (3)	0.36844 (7)	0.34499 (16)	0.0207 (4)	
C3	0.6738 (3)	0.34996 (7)	0.48640 (16)	0.0188 (4)	
C4	0.8923 (2)	0.37633 (8)	0.51184 (18)	0.0208 (4)	
C5	1.0043 (3)	0.35815 (8)	0.64297 (17)	0.0260 (5)	
C6	0.9061 (3)	0.31220 (8)	0.74665 (18)	0.0266 (5)	
C7	0.6930 (3)	0.28560 (8)	0.71952 (17)	0.0244 (5)	
C8	0.5774 (3)	0.30421 (7)	0.59086 (16)	0.0204 (4)	
C9	0.5027 (2)	0.45884 (7)	0.14700 (16)	0.0214 (4)	
C10	0.6300 (3)	0.43023 (9)	0.01323 (18)	0.0313 (5)	
C11	0.5134 (3)	0.45859 (9)	−0.12729 (18)	0.0387 (6)	
H1a	0.382262	0.478981	0.454451	0.0228*	
H2a	0.392563	0.362122	0.359869	0.0249*	
H2b	0.594229	0.335034	0.267886	0.0249*	
H5	1.151137	0.37767	0.661739	0.0312*	
H6	0.984963	0.299065	0.836016	0.0319*	
H8	0.429374	0.285198	0.574125	0.0245*	
H9	0.361614	0.434259	0.134648	0.0257*	
H10a	0.626351	0.378021	0.013612	0.0375*	
H10b	0.781744	0.447839	0.016012	0.0375*	
H11a	0.368381	0.436182	−0.136609	0.0464*	
H11b	0.597131	0.443924	−0.213654	0.0464*	
H1o	0.893 (3)	0.4356 (10)	0.347 (2)	0.0334*	

### Atomic displacement parameters (Å^2^)

**Table:**

	*U*^11^	*U*^22^	*U*^33^	*U*^12^	*U*^13^	*U*^23^
Cl1	0.0514 (3)	0.0328 (2)	0.0254 (2)	−0.00039 (19)	0.00732 (18)	0.00639 (15)
O1	0.0191 (5)	0.0233 (5)	0.0410 (6)	−0.0026 (4)	0.0007 (5)	0.0049 (5)
N1	0.0231 (6)	0.0132 (6)	0.0208 (6)	0.0018 (5)	0.0003 (5)	0.0012 (5)
C1	0.0188 (10)	0.0153 (9)	0.0231 (10)	−0.0004 (8)	0	0
C2	0.0217 (7)	0.0140 (6)	0.0264 (8)	−0.0016 (6)	−0.0020 (7)	−0.0004 (6)
C3	0.0200 (7)	0.0116 (6)	0.0249 (8)	0.0028 (6)	−0.0009 (6)	−0.0019 (6)
C4	0.0173 (7)	0.0150 (6)	0.0300 (9)	0.0027 (5)	0.0017 (6)	−0.0035 (6)
C5	0.0200 (8)	0.0239 (7)	0.0341 (9)	0.0039 (6)	−0.0053 (7)	−0.0055 (7)
C6	0.0314 (9)	0.0230 (7)	0.0253 (8)	0.0095 (7)	−0.0050 (7)	−0.0058 (6)
C7	0.0339 (9)	0.0179 (7)	0.0213 (8)	0.0045 (7)	0.0050 (7)	−0.0005 (6)
C8	0.0204 (7)	0.0142 (6)	0.0267 (7)	0.0010 (6)	0.0021 (6)	−0.0023 (6)
C9	0.0246 (8)	0.0177 (8)	0.0218 (7)	0.0029 (5)	−0.0014 (6)	−0.0014 (6)
C10	0.0446 (10)	0.0239 (8)	0.0253 (9)	0.0088 (7)	0.0027 (8)	−0.0020 (7)
C11	0.0611 (13)	0.0322 (10)	0.0229 (8)	0.0134 (8)	−0.0012 (9)	−0.0039 (7)

### Geometric parameters (Å, °)

**Table:**

Cl1—C7	1.7441 (16)	C5—H5	0.96
O1—C4	1.3535 (19)	C6—C7	1.381 (2)
O1—H1o	0.91 (2)	C6—H6	0.96
N1—C1	1.4850 (11)	C7—C8	1.386 (2)
N1—C2	1.4761 (16)	C8—H8	0.96
N1—C9	1.4697 (17)	C9—C9^i^	1.5138 (19)
C1—H1a	0.96	C9—C10	1.514 (2)
C1—H1a^i^	0.96	C9—H9	0.96
C2—C3	1.503 (2)	C10—C11	1.531 (2)
C2—H2a	0.96	C10—H10a	0.96
C2—H2b	0.96	C10—H10b	0.96
C3—C4	1.406 (2)	C11—C11^i^	1.531 (2)
C3—C8	1.384 (2)	C11—H11a	0.96
C4—C5	1.393 (2)	C11—H11b	0.96
C5—C6	1.386 (2)		
			
C4—O1—H1o	106.9 (12)	C5—C6—H6	120.4925
C1—N1—C2	113.70 (8)	C7—C6—H6	120.4933
C1—N1—C9	105.56 (8)	Cl1—C7—C6	119.74 (12)
C2—N1—C9	112.50 (9)	Cl1—C7—C8	119.24 (12)
N1—C1—N1^i^	105.74 (8)	C6—C7—C8	121.01 (14)
N1—C1—H1a	109.4709	C3—C8—C7	120.52 (14)
N1—C1—H1a^i^	109.4712	C3—C8—H8	119.7416
N1^i^—C1—H1a	109.4713	C7—C8—H8	119.7416
N1^i^—C1—H1a^i^	109.4709	N1—C9—C9^i^	101.45 (10)
H1a—C1—H1a^i^	112.9619	N1—C9—C10	117.56 (12)
N1—C2—C3	112.19 (11)	N1—C9—H9	110.2149
N1—C2—H2a	109.4717	C9^i^—C9—C10	110.98 (12)
N1—C2—H2b	109.4705	C9^i^—C9—H9	116.8873
C3—C2—H2a	109.4714	C10—C9—H9	100.538
C3—C2—H2b	109.4717	C9—C10—C11	107.90 (14)
H2a—C2—H2b	106.6084	C9—C10—H10a	109.4715
C2—C3—C4	120.58 (13)	C9—C10—H10b	109.4705
C2—C3—C8	120.48 (13)	C11—C10—H10a	109.4721
C4—C3—C8	118.88 (14)	C11—C10—H10b	109.4708
O1—C4—C3	121.52 (14)	H10a—C10—H10b	110.9991
O1—C4—C5	118.68 (13)	C10—C11—C11^i^	112.71 (13)
C3—C4—C5	119.80 (14)	C10—C11—H11a	109.4707
C4—C5—C6	120.75 (14)	C10—C11—H11b	109.4709
C4—C5—H5	119.6278	C11^i^—C11—H11a	109.472
C6—C5—H5	119.6266	C11^i^—C11—H11b	109.4712
C5—C6—C7	119.01 (15)	H11a—C11—H11b	106.0299
			
?—?—?—?	?		

Symmetry codes: (i) −*x*+1, −*y*+1, *z*.

### Hydrogen-bond geometry (Å, °)

**Table:**

*D*—H···*A*	*D*—H	H···*A*	*D*···*A*	*D*—H···*A*
C1—H1a···O1^ii^	0.95	2.56	3.3398 (11)	137.58
O1—H1o···N1	0.91 (2)	1.83 (2)	2.6515 (13)	149.3 (18)

Symmetry codes: (ii) *x*−1, *y*, *z*.
